# New Application of an Old Drug: Anti-Diabetic Properties of Phloroglucinol

**DOI:** 10.3390/ijms251910291

**Published:** 2024-09-24

**Authors:** Krzysztof Drygalski, Mateusz Maciejczyk, Urszula Miksza, Andrzej Ustymowicz, Joanna Godzień, Angelika Buczyńska, Andrzej Chomentowski, Iga Walczak, Karolina Pietrowska, Julia Siemińska, Cezary Pawlukianiec, Przemysław Czajkowski, Joanna Fiedorczuk, Monika Moroz, Beata Modzelewska, Anna Zalewska, Barbara Kutryb-Zając, Tomasz Kleszczewski, Michał Ciborowski, Hady Razak Hady, Marc Foretz, Edyta Adamska-Patruno

**Affiliations:** 1 Department of Hypertension and Diabetology, Medical University of Gdansk, 80-214 Gdansk, Poland; 2 Department of Hygiene, Epidemiology and Ergonomics, Medical University of Bialystok, 15-089 Bialystok, Poland; mat.maciejczyk@gmail.com; 3 Clinical Research Support Centre, Medical University of Bialystok, 15-089 Bialystok, Poland; urszula.miksza@umb.edu.pl (U.M.); przemyslaw.czajkowski@umb.edu.pl (P.C.); joanna.fiedorczuk@umb.edu.pl (J.F.); monika.moroz@umb.edu.pl (M.M.); edyta.adamska-patruno@umb.edu.pl (E.A.-P.); 4 Department of Radiology, Medical University of Bialystok, 15-089 Bialystok, Poland; 5 Clinical Research Centre, Medical University of Bialystok, 15-089 Bialystok, Polandangelika.buczynska@umb.edu.pl (A.B.); karolina.pietrowska@umb.edu.pl (K.P.); julia.sieminska@umb.edu.pl (J.S.); michal.ciborowski@umb.edu.pl (M.C.); 6 Department of Biophysics, Medical University of Bialystok, 15-089 Bialystok, Poland; andrzej.chomentowski@sd.umb.edu.pl (A.C.); beata.modzelewska@umb.edu.pl (B.M.); tomasz.kleszczewski@umb.edu.pl (T.K.); 7 Department of Biochemistry, Medical University of Gdansk, 80-214 Gdansk, Poland; igawalczak@gumed.edu.pl (I.W.); b.kutryb-zajac@gumed.edu.pl (B.K.-Z.); 8 Students Scientific Club “Biochemistry of Civilization Diseases”, Department of Hygiene, Epidemiology and Ergonomics, Medical University of Bialystok, 15-089 Bialystok, Poland; 9 Experimental Dentistry Laboratory, Medical University of Bialystok, 15-089 Bialystok, Poland; stom.dos@umb.edu.pl; 10 Clinical Department of General and Endocrine Surgery, Medical University of Bialystok, 15-089 Bialystok, Poland; 11 Institut Cochin, Université Paris Cité, CNRS, INSERM, F-75014 Paris, France; marc.foretz@inserm.fr

**Keywords:** phloroglucinol, NAFLD, diabetes, oxidative stress, insulin resistance, liver steatosis, anti-spasmodic, lipid metabolism

## Abstract

Phloroglucinol (PHG), an analgesic and spasmolytic drug, shows promise in preventing high-fat-diet (HFD)-induced non-alcoholic fatty liver disease (NAFLD) and insulin resistance. In Wistar rats, 10 weeks of PHG treatment did not prevent HFD-induced weight gain but significantly mitigated fasting hyperglycemia, impaired insulin responses, and liver steatosis. This protective effect was not linked to hepatic lipogenesis or AMP-activated protein kinase (AMPK) activation. Instead, PHG improved mitochondrial function by reducing oxidative stress, enhancing ATP production, and increasing anti-oxidant enzyme activity. PHG also relaxed gastric smooth muscles via potassium channel activation and nitric oxide (NO) signaling, potentially delaying gastric emptying. A pilot intervention in pre-diabetic men confirmed PHG’s efficacy in improving postprandial glycemic control and altering lipid metabolism. These findings suggest PHG as a potential therapeutic for NAFLD and insulin resistance, acting through mechanisms involving mitochondrial protection, anti-oxidant activity, and gastric motility modulation. Further clinical evaluation is warranted to explore PHG’s full therapeutic potential.

## 1. Introduction

The increasing worldwide rates of overweight and obesity constitute a growing medical issue and a major public health burden [1]. The unprecedented access to high-caloric foods combined with a simultaneous reduction in physical activity and energy expenditure is leading to a long-lasting positive energy balance, resulting in weight gain. An increased body weight and high-fat diet lead to fat accumulation in peripheral tissues, such as the liver, causing non-alcoholic fatty liver disease (NAFLD). Hepatocytes’ steatosis makes them less responsive to insulin and stimulates insulin resistance, as well as oxidative stress and mitochondrial damage [2,3,4,5]. To compensate insulin resistance in peripheral insulin-dependent tissues, the pancreas produces more insulin to maintain normal blood glucose levels. Over time, this compensatory mechanism may become insufficient, leading to elevated blood sugar levels and the eventual development of type 2 diabetes (T2DM). On the other hand, as an anabolic, insulin promotes the storage of fat, and when its levels are persistently high due to insulin resistance, it can lead to increased fat accumulation, particularly in visceral adipose tissue. This creates a vicious cycle, as excess body fat, especially visceral fat, further exacerbates insulin resistance, resulting in difficulty in losing weight or weight gain [6].

Additionally, insulin resistance hampers the body’s ability to use glucose effectively for energy, often resulting in fatigue, postprandial hypoglycemia, and reduced physical activity, which further contributes to weight management issues. Addressing insulin resistance through lifestyle changes such as dietary interventions, increased physical activity, and weight management is essential for preventing its adverse health outcomes but is often fruitless [7,8]. That is why patients with insulin resistance or who are pre-diabetic seek pharmacological help. 

The most widely used anti-diabetic drug that effectively reduces insulin resistance is metformin. By activating AMP-activated protein kinase (AMPK), metformin enhances cellular glucose uptake and utilization, particularly in muscle tissue, while simultaneously inhibiting hepatic glucose production [9]. This dual action helps lower blood glucose levels and improves insulin sensitivity. Additionally, metformin’s influence on AMPK contributes to reduced fat accumulation and improved lipid metabolism, which further mitigates insulin resistance. Its efficacy, safety profile, and additional benefits, such as weight stabilization and potential cardiovascular protection, make metformin a cornerstone in the management of insulin resistance and T2DM [10,11,12].

Using a drug repurposing approach, we show that phloroglucinol has anti-diabetic and anti-oxidative properties and could be used in the treatment of non-alcoholic fatty liver disease (NAFLD).

However, in the last decade, numerous new compounds have been tested to treat insulin resistance and liver steatosis. Beginning with the resveratrol boom in the early XXI century, multiple polyphenolic substances, such as curcumin or enterolactone, have shown anti-diabetic, anti-oxidant, or anti-inflammatory properties in preclinical studies [13,14,15,16,17]. However, despite promising results in some cases, all these attempts failed to be introduced into clinical practice due to the poor pharmacokinetics of polyphenols and very low availability in humans [18]. That is why, in our project, we focused on phloroglucinol (PHG) (benzene-1,3,5-triol), an organic compound from the phenol group similar to resveratrol but characterized with good availability in vivo and anti-oxidative, anti-inflammatory and anti-glycating properties [19,20]. Furthermore, being used for a long time as a generic drug, PHG is well tolerated and affordable, which makes it a promising candidate for drug repurposing in the treatment of NAFLD and insulin resistance. 

In the following study, we describe the anti-diabetic properties of PHG, an antispasmodic and analgesic drug also found in *Ecklonia cava* extracts. It reduced insulin resistance, liver steatosis, and mitochondrial oxidative stress in rat models of NAFLD, as well as ameliorated postprandial glucose tolerance and lipid metabolism in pre-diabetic men. As an example of translatory medicine, our study shows that drug repurposing may provide an efficient approach to discovering new therapeutic uses for existing medications.

## 2. Results

### 2.1. PHG Protects Wistar Rats from High-Fat-Diet-Induced NAFLD and Insulin Resistance

**Figure 1:**
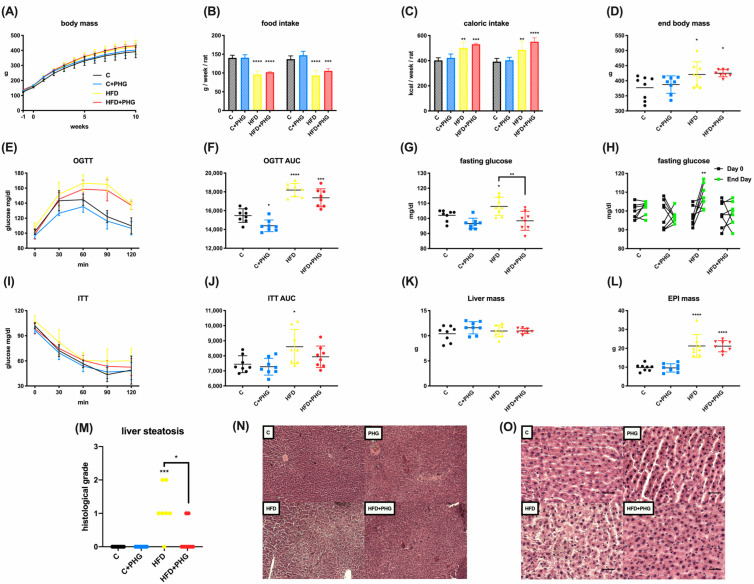
The effect of PHG treatment on insulin resistance in the NAFLD model. The effect of ten−week HFD feeding of Wistar rats on body weight (**A**). Mean food (**B**) and caloric (**C**) intake. End body weight (**D**). The effect of ten-week HFD and PHG treatment on oral glucose tolerance test (OGTT) (**E**), OGTT AUC (**F**), end-day fasting glucose (**G**), change in fasting glucose during the experiment (**H**), insulin tolerance test (ITT) (**I**), ITT AUC (**J**), liver mass (**K**), epididymal fat mass (**L**), histological liver steatosis (**M**–**O**). n = 32; * *p* < 0.05, ** *p* < 0.01, *** *p* < 0.001, **** *p* < 0.0001. If not indicated otherwise * refers to control vs. particular group. For H * refers to day 0 vs. end day. Scale bar refers to 50 μm.

Ten weeks of a high-fat diet (HFD) led to a significant increase in mean caloric intake, resulting in higher body weight, liver steatosis, and insulin resistance in Wistar rats. Despite PHG treatment not preventing HFD-induced weight gain, it was sufficient to prevent its metabolic consequences, such as increased fasting glucose or impaired response to insulin in an insulin tolerance test (ITT) (Figure 1A,G,I,J). Moreover, PHG also ameliorated glycemic control in control diet rats, as seen in the change in the oral glucose tolerance test (OGTT) AUC (Figure 1E). These anti-diabetic effects of PHG treatment in the HFD group could be associated with a significant decrease in the histological grade of liver steatosis (Figure 1N,O). 

### 2.2. The Effect of PHG Is Not Related to Hepatic Lipogenesis or AMPK Mediated

**Figure 2:**
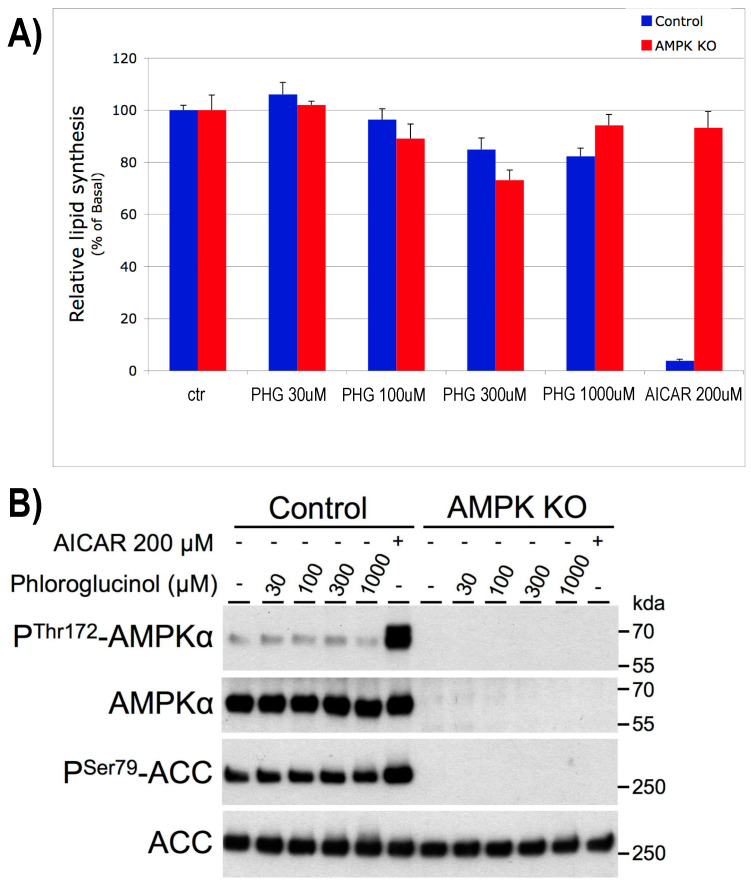
Effect of phloroglucinol on lipid synthesis in control and AMPK-deficient primary mouse hepatocytes. The effect of PHG and AICAR on de novo lipid synthesis (**A**) and phosphorylation of AMPKα at Thr172, and ACC at Ser79. A representative picture of the Western blotting membrane is shown (**B**). n = 3.

One of the possible explanations for the results observed in Wistar rats could be due to AMPK activation, resulting in decreased lipogenesis in the liver. To assay that potential, the effect of PHG on lipogenesis was assessed via the incorporation of 14C-acetate tracer into the lipids in control and AMPK KO mice hepatocytes during a 3 h treatment. In both the control and AMPK-deficient hepatocytes, PHG had no significant effect on lipid synthesis up to 100 µM. At very high concentrations (300 µM and 1 mM), lipid synthesis was inhibited by barely 20%, similarly in control and AMPK KO hepatocytes (Figure 2A). In contrast, the treatment of hepatocytes with AICAR at 200 µM, a well-known AMPK activator, inhibited lipogenesis by >95% in control, and this effect was fully blunted in AMPK KO hepatocytes, demonstrating that AICAR inhibits lipogenesis in an AMPK-dependent manner. Western blot analysis showed that treatment of control hepatocytes with PHG for 3 h did not increase AMPK phosphorylation on Thr172 or the phosphorylation of its target ACC on Ser79, even at concentrations as high as 1 mM. In contrast, AICAR at 200 µM induced robust phosphorylation of AMPK and ACC (Figure 2B). In conclusion, it seems clear that in hepatocytes, PHG does not affect lipid synthesis (<20% at high concentrations >300 µM), and it is not an AMPK activator, even at concentrations as high as 1 mM. Thus, the observed decrease in liver steatosis in rats in response to PHG does not seem to be mediated by lipogenesis inhibition or by AMPK activation in hepatocytes.

### 2.3. PHG Protects Mitochondria from Oxidative Stress

**Figure 3:**
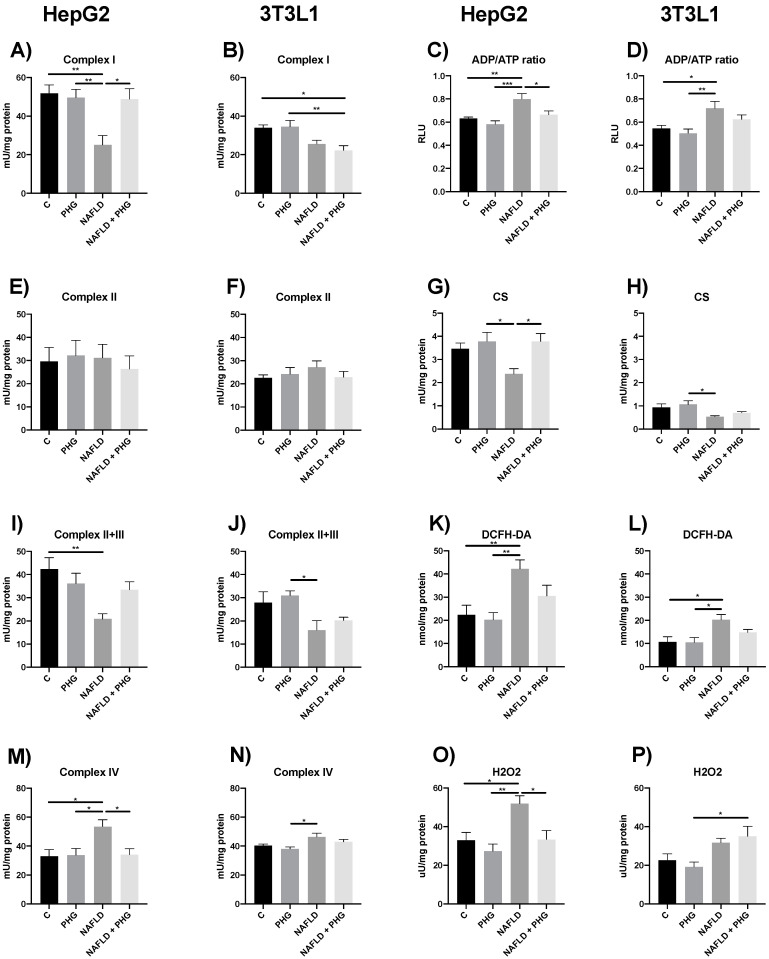
The effect of PHG on mitochondrial function. The effect of PHG on mitochondrial complex I (**A**,**B**), complex II (**E**,**F**), complexes II+III (**I**,**J**) and complex IV (**M**,**N**) activity, ATP/ADP ratio (**C**,**D**), CS (**G**,**H**), DCFH-DA (**K**,**L**) and H_2_O_2_ production (**O**,**P**) in mitochondria isolated from HepG2 and 3T3L1 model of NAFLD respectively. n = 3. * *p* < 0.05, ** *p* < 0.01, *** *p* < 0.001.

Since the effect of PHG on liver steatosis and insulin resistance was not AMPK mediated or related to lipid synthesis, we focused on its potential effect on mitochondrial function, as they are responsible for lipid oxidation. To assess that, we used two cell culture models: HepG2 and differentiated 3T3-L1, which were exposed to a 0.75 mM palmitic acid (PA) medium for 24 h to induce steatosis and oxidative stress. The exposure to PA significantly inhibited the activity of both complexes I and II + III, which was partly reversed in the presence of PHG. The reduced activity of respiratory complexes was accompanied by increased ROS production and an ADP/ATP ratio in the group exposed to PA (Figure 3). In summary, these results indicate PA-induced impairment of mitochondrial function, manifested by reduced energy production and increased electron leakage, leading to intensified free radical formation. 

**Figure 4:**
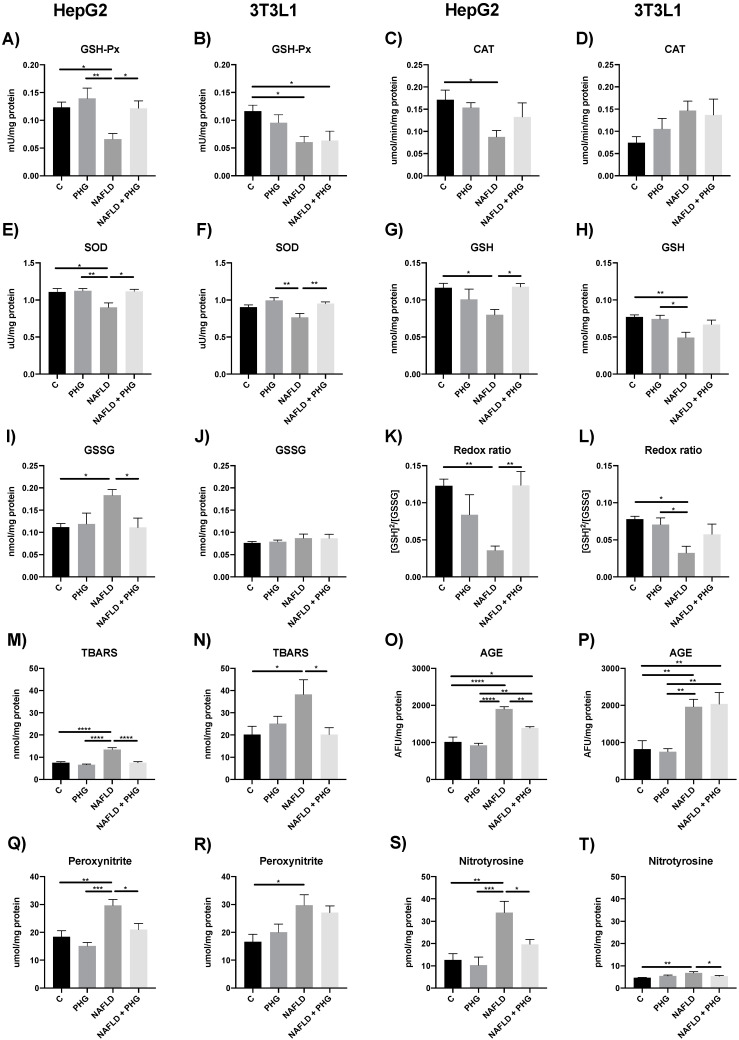
The effect of PHG on mitochondrial enzymatic anti-oxidants, redox ratio and oxidative damage. The effect of PHG on mitochondrial activity of GSH-Px (**A**,**B**), CAT (**C**,**D**), SOD (**E**,**F**), concertation of GSH (**G**,**H**) and GSSG (**I**,**J**), redox ratio (**K**,**L**), TBARS (**M**,**N**), AGEs (**O**,**P**), peroxynitrite (**Q**,**R**) and nitrotyrosine production (**S**,**T**) in mitochondria isolated from HepG2 and 3T3L1 model of NAFLD respectively. n = 3. * *p* < 0.05, ** *p* < 0.01, *** *p* < 0.001, **** *p* < 0.0001.

**Figure 5:**
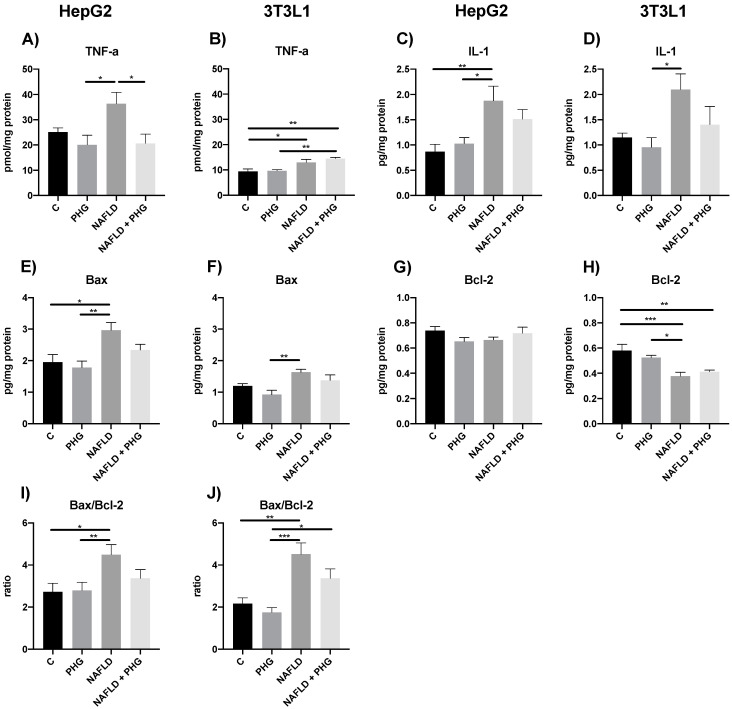
The effect of PHG on inflammation and apoptosis. The effect of PHG on media TNFα (**A**,**B**) and Il-1 (**C**,**D**) concentration, as well as mitochondrial expression of Bax (**E**,**F**), Bcl-2 (**G**,**H**) and Bax/Bcl-2 ratio (**I**,**J**) in mitochondria isolated from HepG2 and 3T3L1 model of NAFLD respectively. n = 3. * *p* < 0.05, ** *p* < 0.01, *** *p* < 0.001.

Unsurprisingly, the increased ROS production resulted in elevated mitochondrial damage, which could have been prevented when PHG was added to the culture media. The drug normalized the concentration of both reduced and oxidized glutathione and restored a control redox ratio. Furthermore, PHG increased the activity of enzymatic anti-oxidants: GSH-Px and SOD in mitochondria, which was inhibited by PA (Figure 4). Taken together, our findings suggest that PHG normalizes mitochondrial function and protects them from oxidative damage. Its effect seems to be multifactorial, affecting cellular steatosis, mitochondrial complexes’ activity, and enzymatic anti-oxidants. As a downstream effect of PHG protective activity, we observed a decrease in TNF-a and IL-1 concentrations in media and the normalization of key regulators of apoptosis: Bax and Bcl-2 in both analyzed cell lines (Figure 5).

### 2.4. PHG Relaxes Gastric Smooth Muscles

**Figure 6:**
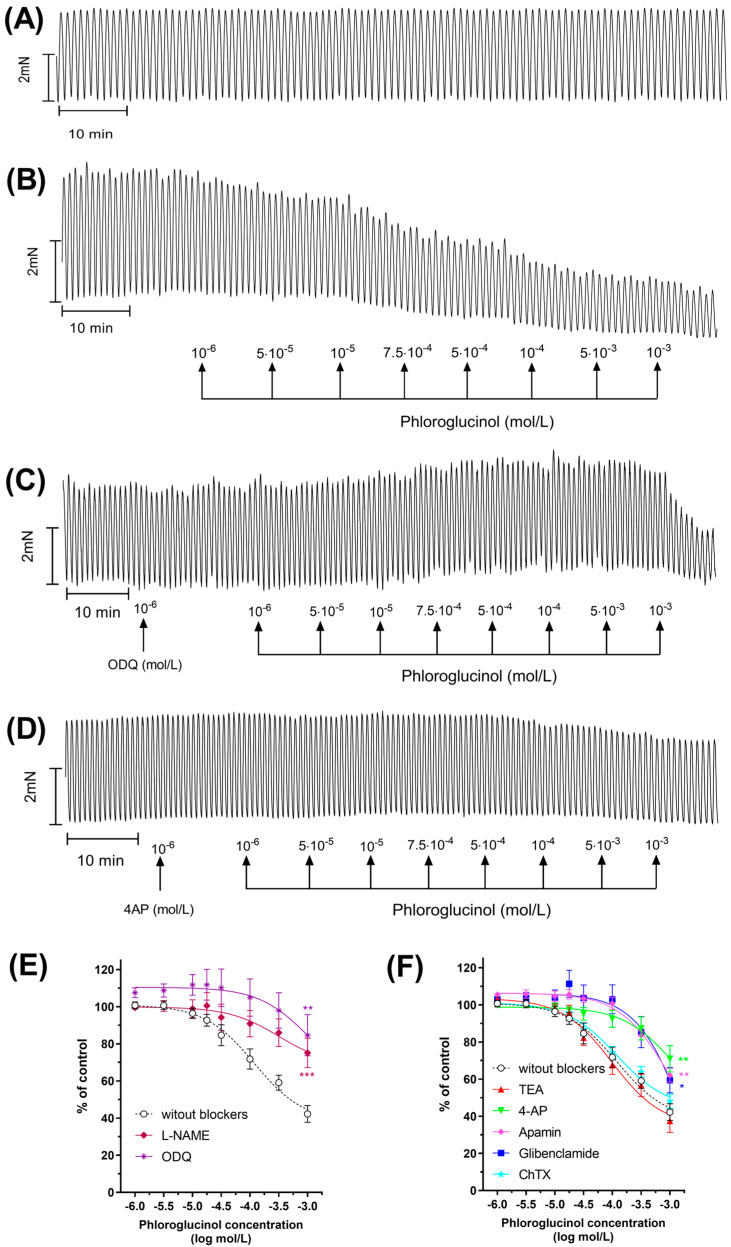
PHG relaxes gastric smooth muscles. A typical recording of carbachol-induced contractile activity of the human gastric strips (**A**) and the effect of cumulatively administered PHG (range 10^−6^–10^−3^ mol/L) (**B**). Representative recording for the blocking effect of ODQ (**C**) and 4-AP (**D**) pre-treatment on PHG-induced muscle relaxation. Effects of PHG after preincubation with L-NAME, ODQ (**E**); and TEA, 4-AP, ChTX, glibenclamide, or apamin (**F**) on the gastric strips, as measured by AUC. Each point represents the mean ± SEM of values obtained from individual gastric strips (n = 10) from ten different patients. Contractions of the gastric strips before phloroglucinol were treated as controls. * *p* < 0.05, ** *p* < 0.01, *** *p* < 0.001 versus PHG alone.

Since PHG is registered as an analgesic and spasmolytic drug, another possible explanation for its effects on glycaemic control could be gastric muscle relaxation, resulting in delayed gastric emptying and food passages. Interestingly, despite PHG being used as a spasmolytic drug for decades, the molecular mechanism of its action has not been clearly explained so far. Thus, to answer whether PHG affects gastric smooth muscle contractility and to look for a molecular explanation of its effects, we established an ex vivo gastric smooth muscle bath culture. In the muscle strips that were isolated from the upper half of the human stomach, carbachol, in the concentration of 10^−6^ mol/L, promoted a noticeable and stable muscle contractility (Figure 6A). Typical tracings show the response of gastric smooth muscle strips when cumulatively applied to PHG (Figure 6B).

Pre-treatment with apamin, a specific blocker of the SK_Ca_ channel, altered PHG-induced relaxation significantly at all concentrations of the drug. Similarly, preincubation with a K_ATP_ channel blocker, glibenclamide, or a K_v_ channel blocker, 4-AP, caused considerable inhibition of muscle contractions (Figure 6D). A statistically significant shift to the right from the concentration–response curve for PHG observed after preincubation with both apamin, glibenclamide, or 4-AP showed a noncompetitive antagonism of tissue response to high efficacy (receptor reserve present). However, PHG-induced relaxation was not inhibited by non-selective K+ channel inhibitors TEA and ChTX, inhibitors of BK_Ca_ and IK_Ca_, respectively (Figure 6F).

Since NO, as one of the most significant NANC neurotransmitters in the GI tract, controls smooth muscle relaxation by cGMP-dependent or -independent mechanisms, we decided to test its relationship with PHG. The data presented clearly show that after preincubation with a guanylate cyclase blocker—ODQ—a statistically significant decrease in AUC was observed at all PHG concentrations (Figure 6C). In addition, PHG-induced relaxation was blocked by NO inhibition with L-NAME. Both effects were accompanied by a statistically significant shift to the right of the concentration–response curves for PHG, indicating that gastric strip relaxation caused by it is also related to NO production (Figure 6E). All Log EC50 and Emax data are summarized in Table 1. These results indicate that PHG relaxes gastric smooth muscles by activating certain types of potassium channels, SK_Ca_, K_ATP_, and K_v_ channels, as well as through NO signaling.

### 2.5. PHG Ameliorates Postprandial Glucose Tolerance and Calorimetry

**Figure 7:**
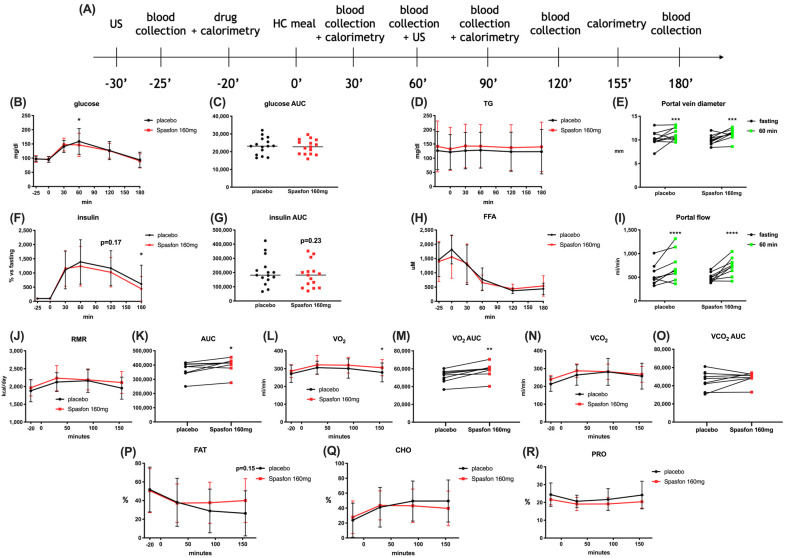
The effect of single dose Spasfon 160 mg on postprandial glycemic control and calorimetry in pre-diabetic male volunteers. Meal test and IMP intervention visit design (**A**). Postprandial glucose level (**B**), glucose AUC (**C**), triglyceride (**D**), insulin (**F**), insulin AUC (**G**), free fatty acid level (**H**). Postprandial change in portal vein diameter (**E**) and portal flow (**I**). Postprandial energy expenditure (resting metabolic rate (RMR)) (**J**), RMR AUC (**K**), oxygen consumption (**L**), oxygen consumption AUC (**M**), carbon dioxide production (**N**), carbon dioxide production AUC (**O**), percentage of fat (**P**), carbohydrates (**Q**) and protein (**R**) in substrates utilization. For biochemical assays n = 15, for calorimetry and US examination n = 10. * *p* < 0.05, ** *p* < 0.01, *** *p* < 0.001, **** *p* < 0.0001 placebo vs. Spasfon 160 mg. For portal diameter and flow * refers to fasting vs. postprandial.

Because acute exposure to PHG induced gastric muscle relaxation, we wanted to verify if this effect can affect postprandial glycemic control, portal flow, and calorimetry in humans. To measure that, we recruited 15 pre-diabetic male volunteers to conduct a pilot, double-blinded, single-dosage, crossover intervention. We conducted a challenge test with a high-carbohydrate meal intake, and surprisingly, even after a single dosage of PHG, we observed a significantly lower postprandial peak of glycemia and insulin concentrations (Figure 7B,F). However, it has to be noted that due to high variability between participants, statistically significant changes in postprandial insulin concentrations were observed only if results were expressed as a % change from the baseline (fasting) insulin concentration for each individual. Nevertheless, considering the small group size and acute exposure, it seems promising to assess the anti-diabetic effects of chronic PHG treatment in future clinical trials in pre-diabetic or T2DM patients. Since it is unlikely that a single dosage affected insulin resistance, the observed effect on postprandial glycemic control might support our hypothesis on delayed gastric emptying and food passages. PHG did not affect the portal vein diameter or portal flow (Figure 7E,I). On the other hand, pre-treatment with PHG led to slightly increased postprandial energy expenditure and oxygen consumption with a pronounced yet not significant trend toward lipid oxidation (Figure 7J–R).

### 2.6. PHG Affects Postprandial Lipid Metabolism

**Figure 8:**
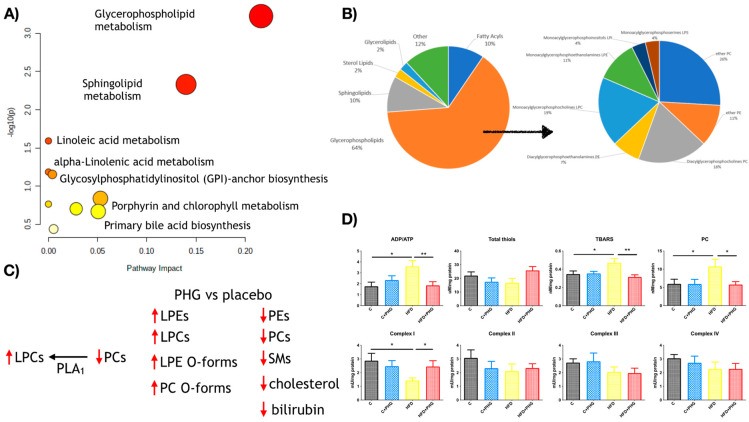
The effect of PHG on lipid metabolism**.** Pathway analysis of the effects of PHG on postprandial metabolomics (**A**) and the classification of significantly changed metabolites (**B**). Main changes in serum metabolites induced by a single-dosage PHG administration in pre-diabetic men (**C**). The effects of ten-week PHG treatment on mitochondrial activity and oxidative damage in the liver of high-fat-fed Wistar rats (**D**). For human metabolomics data n = 10, for Wistar NAFLD model n = 32; * *p* < 0.05, ** *p* < 0.01.

To screen for possible metabolic effects of PHG, we applied untargeted metabolomics in plasma samples collected during the high-carbohydrate meal test. Pathway analysis was performed for 29 unique annotated metabolites. The analysis revealed that the treatment highly affected the glycerophospholipid, sphingolipid, and linoleic acid metabolism (Figure 8A). Almost all metabolites differentiating between placebos and the drug in postprandial metabolomics were classified as lipid: 64% belonged to the glycerophospholipids class, 10% were sphingolipids, 10% were fatty acyls, and 2% were sterol lipids as well as glycerophospholipids (Figure 8B). Within fatty acyls, carnitines (CAR 6:0, CAR 12:1, and CAR 14:1) decreased after the drug administration, while aminopentanoic acid increased. Lysophosphatidylcholines (LPC 18:0, LPC 18:1, LPC 18:2 and LPC 18:3) increased after drug administration similarly to ether-lysophosphatidylcholines (LPC O-16:0, LPC O-18:2/LPC P-18:1 and LPC O-18:2/LPC P-18:1). In contrast, phosphatidylcholines and ether-phosphatidylcholines behaved differently: most phosphatidylcholines (PC 36:4, PC 37:4, PC 38:5, PC 40:7) decreased, while the majority of ether-phosphatidylcholines (PC O-34:2/PC P-34:1, PC O-38:67/PC P-38:6 and PC O-21:0) increased after the drug administration. Glycerophosphatidylethanolamines behaved similarly to the glycerophosphocholines: lysophosphatidylethanolamines (LPE 18:1 and LPE 18:3) increased after the drug administration, while diacyl glycerophosphoethanolamines (PE 38:1 and PE 38:4) decreased. Interestingly, all discriminating LPCs and LPEs were annotated as sn-1 forms. Within sphingolipids both sphingomyelins (SM(d33:1), SM(d34:0), SM(d38:2)), and galactosylceramide (d34:1) decreased after drug administration. Finally, only one sterol lipid was found to significantly differ between groups, cholesterol, slightly decreasing in the plasma of patients receiving the drug. Taken together, the metabolomics results suggest that PHG may affect the activity of phospholipase A1 (PLA_1_) since it decreased PCs and increased LPCs. 

Similarly, increased ether forms of PCs and LPCs may confirm its anti-oxidative effects in vivo since these modified phospholipids served as cellular anti-oxidants (Figure 8C). The metabolomic results and postprandial calorimetry seem to be in line with the results observed in the rat model of NAFLD. Where chronic PHG treatment restored mitochondrial complexes’ activities and ATP production in liver mitochondria what was disrupted by HFD-feeding. However, when it was combined with the anti-oxidative properties of PHG, it led to a significant decrease in mitochondrial oxidative damage (Figure 8D).

## 3. Discussion

Drug repurposing offers a promising strategy for discovering new therapeutic applications, leveraging the benefits of reduced development time, cost, and established safety profiles [21,22]. In this study, we explored the repurposing of PHG, a generic drug registered as an analgesic and spasmolytic, for its effects on insulin resistance and liver steatosis in a Wistar rat model of non-alcoholic fatty liver disease (NAFLD) and pre-diabetic men. This translational research approach revealed significant findings on the protective effects of PHG against high-fat-diet (HFD)-induced metabolic disorders.

Our study demonstrated that PHG effectively prevents the development of HFD-induced liver steatosis and insulin resistance in Wistar rats. In contrast to previously published results in C57BL/6J mice, we found that this protective effect of PHG is not associated with AMP-activated protein kinase (AMPK) activation or lipid synthesis pathways in the liver [23]. Instead, PHG exhibits strong anti-oxidative, anti-glycative, and anti-inflammatory properties, which protect mitochondrial function by reducing oxidative stress, restoring ATP production, and maintaining redox balance. This is in line with our previous reports on anti-glycative and anti-oxidant properties of PHG in vitro [19,20]. In this study, we showed that PHG also mitigates oxidative damage at the mitochondrial level. The effect of PHG is more pronounced in insulin-resistant hepatocytes than in adipocytes. Additionally, PHG was found to relax gastric smooth muscles through multiple pathways, including SK_Ca_, K_ATP_, and K_v_ channels, as well as through NO synthesis and signaling. Our observations on gastric track motility should not be surprising since PHG is recommended as an antispasmodic for smooth muscles in the treatment of abdominal pain [24,25,26]. Although it has long been used in clinical practice as an antispasmodic for painful conditions of the urogenital and gastrointestinal (GI) tract, early in vivo studies in anesthetized rats demonstrated that PHG did not affect contractions of the duodenum, ileum, and colon [27]. This may suggest that its action is limited to gastric smooth muscles. Thus, its spasmolytic activity may delay gastric emptying, potentially contributing to a slower food passage and improved postprandial glycemic control. However, to better understand the effect of PHG on GI tract motility and food digestion, it would be necessary to assay the profile of postprandial incretin hormones, which was not possible in our study. On the other hand, in irritable bowel syndrome (IBS), PHG has been shown to reduce glycerol-induced abdominal pain and inhibit colonic phasic contractions without affecting the colonic tone [28].

In a single-dosage crossover intervention study in pre-diabetic men, PHG significantly improved postprandial glycemic control and increased energy expenditure. Untargeted metabolomics pathway analysis revealed that PHG treatment significantly impacts glycerophospholipid, sphingolipid, and linoleic acid metabolism. These changes suggest that PHG may affect the activity of phospholipase A1 (PLA1), as evidenced by decreased PCs and increased LPCs. The rise in ether forms of PCs and LPCs further supports the anti-oxidant effects of PHG in vivo, as these modified phospholipids act as cellular anti-oxidants. Similarly, in a recent study assessing the oxidative stress in retinal pigment epithelium cells, PHG attenuated DNA damage and apoptosis through the inhibition of mitochondrial ROS production [29].

The metabolomics findings align with our observations from the rat NAFLD model, where chronic PHG treatment restored mitochondrial complex activity and ATP production in liver mitochondria disrupted by HFD feeding. This restoration, combined with PHG’s anti-oxidative properties, led to a significant reduction in mitochondrial oxidative damage.

PHG’s ability to prevent liver steatosis and insulin resistance, coupled with its impact on mitochondrial function, underscores its potential as a therapeutic agent for metabolic disorders. The relaxation of gastric smooth muscles and the subsequent delay in gastric emptying likely contribute to improved metabolic outcomes. The enhanced postprandial glycemic control and energy expenditure observed in pre-diabetic men further highlight the translational potential of PHG.

Despite the promising results, the precise molecular mechanisms underlying PHG’s metabolic benefits warrant further investigation. While our study did not find a link between PHG’s effects and AMPK activation, future research should explore other potential targets and pathways that mediate its actions. Additionally, the impact of PHG on other metabolic tissues, such as muscle and adipose tissue, should be examined to fully understand its systemic effects. Nevertheless, our findings indicate that PHG has a multifactorial role in mitigating metabolic dysfunctions associated with NAFLD and insulin resistance. By protecting mitochondrial integrity, enhancing anti-oxidative defenses, and modulating lipid metabolism, PHG emerges as a candidate for drug repurposing in the treatment of T2DM and NAFLD. Further functional and clinical evaluations are needed to confirm its efficacy in broader patient populations.

In conclusion, the repurposing of PHG exemplifies how existing drugs can be leveraged for new therapeutic uses, potentially offering a cost-effective and expedited route to addressing pressing health issues such as NAFLD and insulin resistance. The insights gained from this study pave the way for future investigations into PHG’s mechanisms of action and its potential clinical applications.

Nevertheless, our study has some limitations. We have not assessed the effect of long-term PHG treatment on lipid profiles, hepatic lipid transporters, and lipid metabolism, nor have we assessed the drug’s effect on postprandial incretin signaling. However, our work provides a good starting point for further clinical investigations that could answer at least some of these concerns and verify whether PHG can be used in the treatment of T2DM. 

## 4. Materials and Methods

### 4.1. Animals

Five-week-old Wistar male rats (120–140 g) were purchased from and housed in the Experimental Medicine Center of the Medical University of Bialystok, Bialystok, Poland. All animal experiments and procedures on Wistar rats were performed according to Polish animal care regulations upon acceptance of the Local Ethical Committee in Olsztyn, Poland (22/WPN/2021). The rats were housed under ambient temperatures: 20–24 °C; 12 h of daylight/darkness cycles; 55% ± 10% humidity, two rats per cage. After 5 days of acclimatization, the rats were randomly divided into 4 groups of 8 animals each. The rats were closely monitored for behavioral changes or other adverse events to ensure animal safety. On completing the experiment, the rats were euthanized using CO_2_ with the volatile method, and organs of interest were swiftly retrieved for H&E staining, mitochondria isolation, and molecular assays.

### 4.2. High-Fat Diet (HFD) Treatment

To induce insulin resistance and liver steatosis, the rats in experimental groups were fed with a high-fat diet (Research Diets, New Brunswick, NJ, USA, D12492) composed of 59.8% fats, 20.1% proteins and 20.1% carbohydrates for 10 weeks, while control groups were fed a standard chow diet (Agropol, Motycz, Poland) composed of 10.3% fats, 24.2% proteins and 65.5% carbohydrates for 10 weeks. All animals had ad libitum access to chow and tap water. The control group (C) received a control chow diet and 0,9% NaCl intragastric injection daily, and the PHG group received a control chow diet and 100 mg/kg of PHG intragastric injection daily, the HFD group received a high-fat diet and 0,9% NaCl intragastric injection daily while HFD + PHG group received high-fat diet and 100 mg/kg of PHG intragastric injection daily. The PHG dosage was chosen based on a literature search [23]. 

### 4.3. AMPK Knock-Out Hepatocytes and Lipid Synthesis Assay

The effects of phloroglucinol on lipid synthesis and AMPK activation were assessed in control and AMPK KO hepatocytes isolated from AMPKα1^lox/lox^, α2^lox/lox^ (control) and AMPKα1^lox/lox^, α2^lox/lox^-Alfp-Cre (liver AMPK KO) mice as described previously [30]. The effect of phloroglucinol (at 30, 100, 300, and 1000 µM) on lipogenesis flux was assessed in triplicate via the incorporation of [1-^14^C]-acetate tracer into lipids in control and AMPK KO hepatocytes during a 3 h treatment and compared with AICAR at 200 µM, a well-known AMPK activator, as described previously [31]. AMPK phosphorylation on Thr172, as well as its target, ACC, and phosphorylation on Ser79 were analyzed using standard Western blotting [30]. 

### 4.4. Oral Glucose Tolerance Test (OGTT) and Insulin Tolerance Test (ITT)

Fasting glucose was measured on day 0 and the last day of the experiment. An oral glucose tolerance test (OGTT) was performed on the last day of the experiment upon overnight fasting. All rats received glucose (2 g/kg of body weight) in 0.9% saline solution via intragastric tube. Blood glucose level was measured before glucose administration and 30, 60, 90, and 120 min after. Insulin tolerance test (ITT) was performed 6 days before the end of the experiment upon overnight fasting. All rats received insulin (1 IU/kg of body weight i.p. NovoRapid (Novo Nordisk, Bagsværd, Denmark). Blood glucose level was measured before insulin administration and 30, 60, 90, and 120 min after. All glucose level measurements were performed in tail vein blood using an AccuCheck glucometer (Roche, Basel, Switzerland).

### 4.5. Liver Histology

Freshly dissected liver lobes were fixed in 4% formaldehyde (SigmaAldrich, Warsaw, Poland) overnight and replaced with 70% and 95% ethanol before paraffin embedding and tissue cutting. The slides were stained with hematoxylin and eosin (H&E) and imaged under the light microscope. A total of 2–3 liver sections per animal were assessed in a random order by a blinded pathomorphologist from the Department of Medical Pathomorphology of the Medical University of Bialystok, Poland.

### 4.6. Cell Culture and Mitochondria Isolation

The experimental procedures were conducted on differentiated 3T3-L1, and HepG2 cells seeded on 6-well plates. At the beginning of the experiment, HepG2 cells had a confluence of 90%. 3T3-L1 were differentiated for 8 days, as previously described [32]. To induce the NALFD model, cells were exposed to 0.75 mM of palmitic acid (PA) for 24 h. The PA was dissolved in a growth medium (DMEM, 10%FBS, 1% penicillin/streptomycin) as previously described [15,33]. Cells were divided into 4 experimental groups: control, 100 μM PHG, 0.75 mM PA, 0.75 mM PA^+^ 100μM PHG. After the experiment, cells were homogenized (1:10, *w*/*v*) with a Teflon-on-glass electric homogenizer in an ice-cold mitochondria isolation buffer (250 mM sucrose, 5 mM Tris-HCl, and 2 mM ethylene glycol bis(2-aminoethyl)tetraacetic acid) [34,35]. To prevent proteolysis, protease and phosphatase inhibitors (Roche Diagnostics GmbH, Germany) were added. The homogenate was centrifuged (500× *g*, 10 min, 4 °C), and the resulting supernatant was centrifuged twice at 8000× *g* for 10 min at 4 °C [36]. The mitochondria pellet was resuspended in an isolation buffer and immediately processed [37].

### 4.7. Muscle Relaxation Assay

#### 4.7.1. Sample Processing

The study was conducted under the Helsinki Declaration principles, the International Conference on Harmonisation Guideline for Good Clinical Practice, the laws and regulations of Poland, and with the approval from the Ethical Committee of Medical University of Białystok, Poland (No. R-I-002/304/2018). Tissues were obtained from patients undergoing sleeve gastrectomy due to morbid obesity (n = 10 aged 25–51; BMI 47.31 ± 1.11). The experiment did not affect the course of the operation. Samples were taken from the upper half of the stomach with larger curvature removed during the surgical procedure [38,39]. All patients were carefully informed about the aim and nature of the study before surgery and signed written consent.

#### 4.7.2. Experimental Protocol

After the equilibration period, contractile activity was stimulated using carbachol (10^−6^ mol/L). The contractile activity of strips incubated only with carbachol was considered a control after reaching the plateau. To examine concentration–response relationships, PHG was added cumulatively to the organ chambers (range 10^−6^–10^−3^ mol/L) at 10-min intervals, and the effects were recorded. To verify the role of NOS in the effect of resveratrol 10^−6^ mol/L of N^G^-Methyl-L-arginine (N-Nitroarginine methyl ester, L-NAME), a NO synthase (NOS) blocker and 10^−6^ mol/L ODQ (soluble guanylate cyclase blocker, sGC) were used. Concentration–response curves to PHG were also constructed in the absence and presence of 10^−3^ mol/L tetraethylammonium chloride (TEA), a non-selective K^+^ channel blocker; 10^−9^ mol/L charybdotoxin (ChTX), an inhibitor of high conductance Ca^2+^-dependent (BK_Ca_), intermediate (IK_Ca_) conductance calcium-activated potassium channels (K_Ca_), and slowly inactivating voltage-gated K_Ca_ channels; 10^−3^ mol/L 4-Aminopiridine (4AP)—a voltage-gated K^+^ (K_v_) channel blocker; 10^−6^ mol/L apamin, a small conductance K_Ca_ (SK_Ca_) channel blocker or 10^−6^ mol/L glibenclamide, a K^+^ATP-dependent (K_ATP_) channel blocker. As far as possible, experiments were performed with strips from the same stomach sample and were studied in parallel. Appropriate controls were run under similar experimental conditions obtained from the same patient.

#### 4.7.3. Measurement of Contraction Parameters

Gastric muscle activity was recorded by an isometric force transducer with digital output (BIO-SYS-TECH, Białystok, Poland) and calculated with the DASYLab software unit (version 9.0; Laboratory Data Acquisition System, SuperLogics, Waltham, MA, USA). The area under the curve (AUC) reflected the total change over time, representing the contractile activity of stomach muscle responses before and after the administration of the given drug. The AUC was measured as the area under all recorded contractions over a 10 min interval before the addition of each agonist or antagonist, respectively [40,41]. Values from three to four strips from each sample were averaged at each time point for each drug dose. The AUC of contractions of each muscle strip over a 10 min interval before the addition of PHG was treated as a control (100%).

### 4.8. Single Dose Crossed Intervention

The interventional human study was conducted under the Helsinki Declaration principles, the International Conference on Harmonisation Guideline for Good Clinical Practice, the laws and regulations of Poland, and with the approval from the Ethical Committee of Medical University of Bialystok, Bialystok, Poland (No.APK.002.375.2021). Briefly, 15 male volunteers with impaired fasting glucose (IGF) or impaired glucose tolerance (IGT) were recruited into the randomized, placebo-controlled, double-blinded, single-dose crossover intervention. Taking into consideration the fact that the investigated parameters may be characterized by sexual dimorphism and other factors may have an impact on their concentrations depending on the phase of the menstrual cycle; therefore, only male participants were enrolled in the study [42]. The study included 3 visits: a screening visit and two visits with an intervention. During the screening visit, the inclusion and exclusion criteria were evaluated based on medical history, physical examination, vital signs measurements as well as on laboratory results, including fasting hematology, lipid profile, C-reactive protein (CRP), creatine, aspartate transaminase, alanine transaminase and oral glucose tolerance test (OGTT) conducted accordingly with the American Diabetes Association (ADA) recommendations [43]. OGTT was performed only in individuals without previously known diabetes history; otherwise, only fasting glucose concentration was evaluated. Volunteers enrolled in the study who met inclusion criteria and did not meet exclusion criteria were instructed to maintain their regular lifestyle throughout the whole study participation and to avoid intensive physical activity, intake of alcohol, and food rich in polyphenols (tea, coffee, chocolate, etc.). The main study consisted of two visits during which participants received an investigational medicinal product (IMP) or placebo in a random order, and meal tests were performed. The interventional visits were conducted using a crossover method. Therefore, IMP and placebo interventions were performed in the same individuals, with at least a two-week wash-out period between interventions.

#### 4.8.1. IMP/Placebo

Individuals received both IMP and placebo capsules at different visits in random order. The capsules containing IMP and placebo were prepared in the laboratory by a trained pharmacist just before dispensing to the participant during a meal challenge test. In the gelatin capsule, 160 mg of Spasfon Lyoc (Teva, Tel Awiw, Izrael), containing 160 mg of phloroglucinol dihydrate or a placebo made of starch, was placed. Placebo capsules were visually identical to IMP capsules and were dispensed in identical packaging. Consequently, investigators, site staff, and participants remained blinded throughout the study.

#### 4.8.2. Biochemical Analysis

The blood for measurements of blood glucose, triglycerides, free fatty acids, insulin, and metabolite levels was collected 30, 60, 120, and 180 min after meal intake. The samples were prepared following the manufacturer’s laboratory kit instructions. The plasma glucose concentrations were evaluated using the hexokinase enzymatic method (using Cobas c111, Roche Diagnostics Co., Ltd., Basel, Switzerland). Serum insulin concentrations were assessed using an immunoradiometric assay (INS-Irma, DIA Source S.A., Ottignies-Louvain-la-Neuve, Belgium; using Wallac Wizard 1470 Automatic Gamma Counter, PerkinElmer Life Sciences, Turku, Finland). The hsCRP, creatinine, ALT, AST, lipid profile including total cholesterol, LDL-C, HDL-C, and TG concentrations were assessed by the enzymatic colorimetric assays using commercially available laboratory kits (Cobas c111, Roche Diagnostic Co., Ltd., Basel, Switzerland). Serum free fatty acid level was assessed by the enzymatic colorimetric assay (Zenbio, Durham, NC, USA).

### 4.9. Untargeted Metabolomics

#### 4.9.1. Chemical and Reagents

Ultrapure water was used to prepare all the aqueous solutions and was obtained “in-house” from a Milli-Q Integral 3 system (Millipore, SAS, Molsheim, France). Zomepirac sodium salt, formic acid, LC-MS-grade methanol and acetonitrile, and LC-grade ethanol were purchased from Sigma-Aldrich Chemie GmbH (Steinheim, Germany). 

#### 4.9.2. Sample Preparation

Plasma samples were prepared using the previously described method [44]. On the day of analysis, the samples were thawed on ice. For protein precipitation and metabolite extraction, one plasma sample volume was mixed with three volumes of ice-cold methanol/ethanol (1:1) containing one ppm of zomepirac (internal standard IS). After extraction, samples were stored on ice for 10 min and centrifuged at 21,000× *g* for 20 min at 4 °C. The supernatant was filtered into a glass HPLC vial through a 0.22 μm nylon filter (ThermoFisher Scientific, Waltham, MA, USA). Quality control samples (QCs) were prepared by mixing equal volumes of all raw samples. QCs were treated like the rest of the samples and injected at the beginning of the batch (10 injections) to equilibrate the system and every ten samples to monitor further the analysis’s stability [45]. 

#### 4.9.3. HPLC-MS Analysis

Plasma profiling was performed using 6546 iFunnel ESI-Q-TOF (Agilent Technologies, Santa Clara, CA, USA) coupled with a 1290 Infinity UHPLC system (Agilent Technologies, Santa Clara, CA, USA) with a degasser, binary pump and thermostated autosampler as described in Supplementary Materials Methods.

#### 4.9.4. Metabolite Annotation

The annotation process covered two stages: first, a tentative annotation assignment based on the MS1 data and, second, spectral matching and structural elucidation based on MS/MS data.

Initial tentative identification of the features discriminating between groups based on accurate mass matching was performed using a CEU Mass Mediator (CMM, Madrid, Spain) [46]. CMM uses ionization, adduct formation, and elution order information to rank tentative candidates retrieved from several databases. This led to the tentative assignment of experimental masses to the candidate hits retrieved from the database, which covered accurate mass matching, isotopic distribution determination, and checking of the possible ions and adducts.

To confirm the annotation of the compounds, LC-MS/MS data independent analysis (DIA) was performed. Fragmentation spectra were searched against the Metlin database. In addition, spectra that were not available in the Metlin were manually inspected, and lipid structural elucidation was performed. This was performed using known lipid fragmentation patterns [47,48].

#### 4.9.5. Metabolic Pathway Analysis

Pathway analysis was performed using MetaboAnalyst 5.0 (http://www.metaboanalyst.ca/ accessed on 20 April 2024). Only annotated metabolites significantly discriminating between groups were used for this analysis. The Kyoto Encyclopedia of Genes and Genomes (KEGG) based Homo sapiens library was selected for analysis with a hypergeometric test in over-representation analysis and relative-betweenness centrality in pathway typology analysis (to estimate node importance). Pathway significance was determined from pathway enrichment analysis and based on values for each compound in the dataset.

### 4.10. Molecular Assays

#### 4.10.1. Mitochondrial Activity

A colorimetric assay was used to measure the activity of Complex I (EC 1.6.5.3). 2,6-dichloroindophenol (DCIP) was reduced by electrons accepted from decyl ubiquinol (coenzyme Q_1_), which was reduced after oxidation of NADH by Complex I [34]. The method described by Rustin et al., which is based on colorimetric measurement of succinate-ubiquinone reductase and succinate-cytochrome c reductase activities, was utilized in the analysis of the activities of Complex II and Complex II + III [49]. The activity of cytochrome c oxidase (COX, complex IV) was analyzed colorimetrically at 550 nm wavelength by measuring the oxidation of reduced cytochrome [50]. Citrate synthase (CS) activity was measured colorimetrically using the reaction with 5-thio-2-nitrobenzoic acid generated from 5,5′-dithiobis-2-nitrobenzoic acid during CS synthesis [51]. The ADP/ATP ratio was measured using a bioluminescent method in which luciferase catalysis is the conversion of ATP and luciferin to light. Abcam ADP/ATP Ratio Assay Kit ab65313 was utilized with adherence to the manufacturer’s instructions.

#### 4.10.2. Glutathione Metabolism

The content of oxidized (GSSG) and reduced (GSH) and total glutathione was assessed colorimetrically at a 412 nm wavelength based on the enzymatic reaction between NADPH, 5,5-dithiobis-(2-nitrobenzoic acid) (DTNB), and glutathione reductase GR [52]. For the GSSG determination, samples were thawed and neutralized to pH 6–7 with 1 M chlorhydrol triethanolamine (TEA). Subsequently, samples were incubated with 2-vinylpyridine (to inhibit glutathione oxidation). The concentration of GSH was calculated by comparing the difference between total glutathione and disulfide glutathione levels. The redox ratio was calculated according to the formula [GSH]^2^/[GSSG] [53,54]. The spectrophotometric method described by Paglia and Valentine was used to assess serum glutathione peroxidase (GPx) activity. In this method, organic peroxides are reduced in the presence of NADPH. One unit of GPx activity was defined as the amount of the enzyme needed to catalyze the oxidation of 1 μmol of NADPH for 1 min, and the absorbance was assessed at 340 nm.

#### 4.10.3. Nitrosative Stress

Commercial enzyme-linked immunosorbent assay (ELISA) (Nitrotyrosine ELISA; Immundiagnostik AG, Bensheim, Germany) was used to measure the level of nitrotyrosine. All steps were performed in accordance with the manufacturer’s instructions. The peroxynitrite content was measured using a colorimetric method involving peroxynitrite-mediated nitration of phenol to nitrophenol. The resulting color product exhibited a maximum absorption at the wavelength of 405 nm.

#### 4.10.4. Oxidative Stress

The formation of hydrogen peroxide (H_2_O_2_) was quantified by measuring the increase in fluorescence after the reaction of Amplex Red with H_2_O_2_ in the presence of horseradish peroxidase. The observation was performed at 530/590 nm wavelength [55]. The rate of H_2_O_2_ formation was calculated using a standard curve of H_2_O_2_ stabilized solution. Spectrofluorometric analysis was performed to measure the concentration of advanced glycation end product (AGE) [56,57]. Samples were diluted 1:50 (*v*/*v*) in PBS (0.02 M, pH 7.0), and the intensity of fluorescence was assessed at 440/370 nm in a 96-well microplate spectrophotometer [58]. The results were expressed as fluorescence units (AFU)/mg protein. The activity of both catalase (CAT) and superoxide dismutase (SOD) was determined colorimetrically at 340 nm wavelength by measuring hydrogen peroxide decomposition and inhibition of oxidation of epinephrine to adrenochrome, respectively [59,60]. One unit of CAT activity was defined as an amount of the enzyme that degrades 1 μmol of hydrogen peroxide per minute. One unit of SOD activity was defined as the amount inhibiting the oxidation of epinephrine by 50%.

#### 4.10.5. ELISA

Bax and Bcl-2 expression were assessed using ELISA kits from Cell Biolabs Inc. (San Diego, CA, USA). IL-1 and TNF-α concentrations in media were measured using ELISA kits from EIAab (Wuhan, China). Samples were standardized to assess the total protein concentration using the BCA method. All the assays were performed in triplicate according to manufacturers’ instructions, and the results were averaged.

### 4.11. Data Analysis

Statistical analysis was analyzed using GraphPad Prism 8.3.0 for MacOS (GraphPad Software, La Jolla, CA, USA). All series of data were checked for consistency with a Gaussian distribution following the D’Agostino–Pearson normality test. For muscle contractility results dose-response was determined using one-way ANOVA or the Kruskal–Wallis test, where appropriate. Statistically significant differences between means were determined by Tukey’s post-hoc or a nonparametric Mann–Whitney test, where appropriate. For the rest of the results, the statistical significance was assessed by one-way ANOVA with Tukey’s post-hoc test for multiple comparisons. Values were considered to be statistically significant at *p* < 0.05.

## 5. Conclusions

Our study demonstrates that PHG, a known analgesic and spasmolytic drug, has potent protective effects against HFD-induced NAFLD and insulin resistance. PHG’s efficacy in reducing liver steatosis and improving insulin sensitivity in Wistar rats is attributed to its anti-oxidant properties and mitochondrial protection rather than modulation of hepatic lipogenesis or AMPK activation.

PHG improves mitochondrial function by reducing oxidative stress, restoring ATP production, and enhancing anti-oxidant defenses. Additionally, it relaxes gastric smooth muscles, potentially delaying gastric emptying and thus contributing to better postprandial glycemic control. This mechanism was further supported by a pilot intervention in pre-diabetic men, where PHG administration improved postprandial glucose and insulin profiles and altered lipid metabolism favorably.

The translational potential of PHG as a therapeutic agent for metabolic disorders is significant, given its multifactorial effects on oxidative stress, mitochondrial function, and lipid metabolism. However, further research is necessary to elucidate the precise molecular mechanisms underlying PHG’s metabolic benefits and to evaluate its long-term effects and efficacy in larger clinical populations.

In conclusion, PHG emerges as a promising candidate for repurposing in the treatment of NAFLD and insulin resistance. Its established safety profile and demonstrated metabolic benefits provide a strong foundation for future clinical trials aimed at exploring its full therapeutic potential in metabolic disorders.

## Figures and Tables

**Table 1:** Log EC50 and Emax for PHG on carbachol-induced contractility of the human gastric muscles. The values are mean ± SEM of n = 10 individual gastric strips from different patients. * *p* < 0.05, ** *p* < 0.01, *** *p* < 0.001, **** *p* < 0.0001 versus phloroglucinol alone.

	logEC_50_	*p*-Value	E_max_	*p*-Value
Phloroglucinol alone	−3.92 ± 0.14		42.27 ± 4.54	
Phloroglucinol after preincubation with				
L-NAME	−3.50 ± 0.60 *	0.021	75.15 ± 7.92 ***	*p* < 0.001
ODQ	−2.98 ± 1.17 *	0.0106	84.64 ± 11.12 **	0.0082
4-AP	−2.94 ± 0.57 ****	*p* < 0.0001	70.95 ± 7.06 **	0.0049
ChTX	−3.92 ± 0.11	0.472	48.11 ± 3.84	0.5887
Apamin	−3.00 ± 0.22 ****	*p* < 0.0001	62.11 ± 3.64 **	0.0077
Glibenclamide	−2.65 ± 0.82 ****	*p* < 0.0001	59.47 ± 6.87 *	0.0408
TEA	−4.01 ± 0.14	0.1001	37.49 ± 6.25	0.516

## Data Availability

The data presented in this study are available on request from the corresponding author.

## Supplementary Materials

The following supporting information can be downloaded at: https://www.mdpi.com/article/10.3390/ijms251910291/s1.
