# Supplementary material for: New Application of an Old Drug: Anti-Diabetic Properties of Phloroglucinol
Source: Int J Mol Sci. 2024 Sep 24;25(19):10291. doi: 10.3390/ijms251910291 (PMC11477119; doi:10.3390/ijms251910291)
Supplement: Supplementary file 1 [file ijms-25-10291-s001.zip › ijms-3223193-supplementary.pdf]

Supplementary Methods

Muscle relaxation assay

Sample processing

All surgeries were performed under general anesthesia performed by the same team of anaesthesiologists. Combined general anaesthesia was induced by propofol (1.0-1.5 mg per kg body mass) and opioid analgesic fentanyl (1.0-1.5 ◎g per kg body mass). A non-depolarizing neuromuscular blocking agent cis-atrocurium (0.1-0.2 mg per kg body mass) was also administered at that stage. Maintenance of general anaesthesia was achieved with the volatile method with sevoflurane administered in repetitive doses. Additional doses of cis-atrocurium and opioids were also administered depending on the patient's needs and metabolism.

After removal, specimens were immediately cooled on ice-cold Tyrode's buffer, bubbled with carbogen (95% O_2 +5% CO_2), and immediately transferred to the laboratory where processed as previously described [4142]. Subsequently, the muscle layer was dissected from the gastric wall and cut in the direction of the longitudinal muscles into 10×3×1.5 mm strips. The tissues were attached to an isometric force transducer and placed in 20 ml tissue bath chambers. The temperature of the bath solution was thermostatically maintained at 37°C and continuously bubbled with carbogen. The preparations were allowed to equilibrate for 1h. During this period the passive tension was adjusted to 2 mN. The bath solution was replaced every 20 min of incubation. Before each experiment, strips were activated by 80 mmol/L K^+. Only strips showing a stable response to potassium were used in the experiments [43,44].

Measurement of contraction parameters

All results are expressed as mean ± SEM of experiments performed on muscle strips from different patients or as percentage change [(effect size – baseline contractile response)/baseline contractile response × 100]. When the same protocol was run on two strips from the same gastric specimen, the data were averaged. Mean concentration-response curves to the used drugs were analysed by fitting into a four-parameter logistic equation using non-linear regression. The AUC was evaluated by calculating the integral of the appropriate section of the curve. Concentration-response curves were fitted to the logistic equation using nonlinear regression Y=Bottom+(Top-Bottom)/(1+10^((LogIC50-X)*HillSlope)) (PRISM 6.0, GraphPad Software Inc., San Diego, CA, USA). The maximal response (E_{max}) was expressed as a percentage of the contractile activity before the administration of tested substances, whereas the concentrations of agents that resulted in a half-maximal effect were expressed as -log IC_{50}.

Chemicals

Phloroglucinol (1,3,5-Trihydroxybenzene), Carbamylcholine chloride ((2-Hydroxyethyl) trimethylammonium chloride carbamate; carbachol), N5-(Nitroamidino)-L-2,5-diaminopentanoic acid, N^G-Methyl-L-Arg (Nω-Nitro-L-arginine methyl ester hydrochloride, L-NAME), 1H-[1,2,4]Oxadiazolo [4,3-a]quinoxalin-1-one (ODQ), apamin, charybdotoxin (ChTX), glibenclamide, N,N,N,N-Tetraethylammonium chloride (TEA), 4-Aminopiridine (4AP) were purchased from the Sigma Chemical Company, were purchased from Sigma (St. Louis, MO).

Stock solutions of, carbachol, L-NAME, apamin, ChTX, TEA, 4AP were prepared with bidistilled water, and glibenclamide and ODQ were dissolved in dimethyl sulphoxide (DMSO). Dilution series of PHG were prepared on the day of the experiment and were maintained at room temperature throughout the experiment. The given concentrations were the calculated final concentrations in the organ bath solution. All substances were added directly to the organ bath containing a Tyrode's solution composed of (mmol/L): NaCl 139.6; KCl 2.68; $MgCl_2$ 1.05; NaH_2PO_4 1.33; $CaCl_2$ 1.80; $NaHCO_3$ 25.0; and glucose 5.55.

Intervention visits procedures

The meal test and IMP/placebo intervention procedures are presented in Figure 5-A. The participants arrived at the laboratory at 07.00-08:00 AM on the test day after an overnight 12-hours fast. All individuals were positioned in bed, in a quiet room with thermoneutral conditions (22–25 °C), to rest for at least 30 minutes. After this time the fasting measurement of basal metabolic rate (BMR) and substrate utilization was conducted for 30 min. The fasting portal vein diameter and portal flow were measured by a trained radiologist (the same person for the whole study) using a Doppler ultrasonography method (Toshiba Aplio 400), and the same exam was performed in the 60 minutes of intervention test. The fasting blood samples were collected to determine the concentrations of glucose, triglycerides, free fatty acids, insulin and the baseline metabolites levels. Then, the participants were given a capsule containing the IMP/placebo along with 200 ml of water, and after another 20 minutes, participants received a standardized high-carbohydrate meal (300 ml, Nutricia, Nutridrink Juicy Style strawberry flavour) containing 450 kcal provided by carbohydrates 89%, protein 11%, and fat 0% of energy. The next blood collections were performed at 30, 60, 120 and 180 min after meal intake. Postprandial resting energy expenditure (REE) and substrates utilizations were measured for 30 min each at 60, 120 and 180 min following the meal intake. The energy

expenditure and substrate utilization were evaluated by a computed open-circuit indirect calorimetry method, a noninvasive and recommended method to measure REE - based on the consumption of oxygen and the production of carbon dioxide [49]. The measurements of O2 uptake and CO2 productions were performed by a ventilated canopy Vmax Encore 29n System (Viasys HealthCare, Yorba Linda, CA, USA), one of the most valid instrument for both RMR (Resting Metabolic Rate) and RER (Respiratory Exchange Ratio) assessment [50]. Calibrations were performed with certified CO2 and O2 compositions of gasses before starting the assessments accordingly with manufacturer recommendations, and measurements were standardized by the internal guidelines. The system provided calculated results for energy expenditure and substrate utilization, which were expressed as kcal/day or % values.

Anthropometric measurements

The weight and height were measured in a standardized way [51]. All individuals underwent body weight and body composition analysis, including body fat content (bioelectrical impedance analysis, InBody 270, Biospace, Korea). Body Mass Index (BMI) was calculated as body weight in kilograms divided by the square of height in meters.

HPLC-MS analysis

Plasma profiling was performed using 6546 iFunnel ESI-Q-TOF (Agilent Technologies, Germany) coupled with a 1290 Infinity UHPLC system (Agilent Technologies, Germany) with a degasser, binary pump and thermostated autosampler. One microliter of a sample was injected into a thermostated (60°C) Zorbax Extend-C18 RRHT (2.1×50 mm, 1.8 µm particle size, Agilent Technologies) chromatographic column. The flow rate was 0.6 mL/min with solvent A (deionized water with 0.1% formic acid) and solvent B (acetonitrile with 0.1% formic acid). The chromatographic gradient started at 5% of phase B for the first minute. Next, the mobile phase composition was changed by increasing phase B to 80% (from 1 to 7 min) and 100% (from 7 to 11.5 min). Following that, the system was re-equilibrated by reverting phase composition to initial conditions (5% phase B) in 0.5 min, which was kept from 12 to 15 min. The mass spectrometer was operated in full scan mode. Data were acquired at full scan mode from *m/z* 50 to 1000, separately in positive and negative ion modes at the scan rate of 1.5 scans per second. Nebulizer pressure was set at 52 psi, nozzle voltage at 1000 V, and capillary voltages were set at 3000 and 4000 V in the positive and negative ion mode, respectively. Samples were analyzed in both polarity modes. During all analyses, two reference compounds were used: *m/z* 121.0509 (protonated purine) and *m/z* 922.0098 (protonated hexakis (1H,1H,3H-tetrafluoropropoxy) phosphazine (HP-921)) for positive ionization mode and m/z 112.9856 (proton abstracted trifluoroacetic acid anion) and *m/z* 966.0007 (formate adduct of HP-921) for negative ionization mode. These masses were continuously infused into the system to allow internal constant mass correction during data acquisition. To confirm the annotation of the compounds, LC-MS/MS analysis was carried out, repeating the experiment. The data independent analysis (DIA) was performed using the same chromatographic and spectrometric conditions for the primary analysis. According to the prior determined accurate mass and retention time, ions of interest were targeted by collision-induced dissociation (CID) fragmentation, using a narrow isolation width (approx. 1.3 Da). Collision energies were automatically adjusted for each precursor as a function of its mass and charge.

Data processing

The raw data were processed using recursive analysis in Mass Hunter Profinder software (Agilent B. 10.0). Data was reprocessed twice: 1st reprocessing aimed to create a list of detected signals without major restrictions. Then, the data set obtained through such processing was filtered, and data were curated to remove non-relevant or redundant signals. During this cleaning, blank subtraction was performed: features with averaged signals across the samples expressed ten times greater than 10th percentile ten times greater than the averaged signals in the blank were kept for the analysis. Moreover, redundant singles and known in-source fragments were eliminated during manual curation. A cleaned list was subsequently used for 2nd target reprocessing, where a sophisticated algorithm searched selected ions (1121 for positive ion mode and 1415 for negative ion mode) across all data files. This way, the number of non-relevant, non-biological signals and the number of missing values resulting from reprocessing were reduced. Data were reprocessed considering ions [M+H]+, [M+Na]+, $[M^{+}K]^{+}$, $[CHNO_2]^{+}$ and $[C_2H_2NaO_4]^{+}$ in positive ionization mode and $[M\text{-}H]^{-}$, $[M+HCOO]^{-}$, $[M+Cl]^{-}$, $[C_2HF_3O_2]^{-}$, $[C_3H_2F_3NaO_4]^{-}$ in negative ionization mode. Neutral loss of $[C_2H_4O_2]$ and $[CH_3]$ was used in negative ion mode, while water loss was considered in both polarities. The maximum permitted charge state was double. Alignment was performed based on m/z and RT similarities within the samples. Parameters applied were 0.5% and 0.20 min for the RT window and 20 ppm and 2 mDa for mass tolerance. These were selected based on the assessment of raw data. After 2nd processing, two new matrices were obtained, containing 1136 features in positive and 1428 in negative ion mode. Before any further filtration, data was normalized. Each data set was normalized to the IS signal. Then, Quality Assurance (QA) procedure was performed. Firstly, features present in at least 12 of 16 QCs were selected,

resulting in 1116 in positive and 1415 in negative ion mode. Secondly, the relative standard deviation for signals across QCs was calculated, and values lower than 30% were accepted. This way, matrices were reduced to 945 in positive and 1204 in negative ion mode. Next, filtration was performed across biological samples, keeping only these signals detected across at least 45 out of 60 samples in at least one group, resulting in 926 features in positive and 1195 in negative ion mode. After final curation, matrices contained 869 in positive and 1141 features in negative ion mode. Before data analysis, missing values imputation was performed, employing the k-means nearest neighbour imputation method [54]. The in-house-built script was run in MATLAB (R2015a MathWorks Inc.), and k was set to 3.

Data analysis

Based on the relation between time points and signal intensity for each metabolite, the areas under the curve (AUCs) were calculated using a trapezoid rule in R (version 3.4.3, https://www.R-project.org/). Statistical analysis was performed on obtained AUCs.

Filtered features were then subjected to statistical analysis in which AUCs calculated for patients receiving placebo and drugs were pair-wise compared. P values were computed using paired Mann-Whitney, available in the Mass Profiler Professional software (15.0 Agilent). Multiple testing correction was not applied, with the idea to retrieve as many differencing metabolites as possible. Significantly differentiating features were determined as having a p-value < 0.05. Calculated AUCs were correlated with clinical data. To achieve this Spearman method was implemented. Percentage of change was calculated as (average of signal for drug group – an average of signal for the placebo group)/average of signal for the placebo group, in %, where a positive value indicated an increase in patients receiving the drug compared to the patients receiving placebo. In contrast, a negative value illustrated a decrease in patients receiving the drug. Furthermore, the median, Q1 and Q3 for each significant metabolite were calculated using Excel.
